# Role of miRNA binding site SNPs in candidate genes in a North Indian schizophrenia cohort

**DOI:** 10.1186/1755-8166-7-S1-P91

**Published:** 2014-01-21

**Authors:** Jibin John, Smita N Deshpande, Vishwajit L Nimgaonkar, BK Thelma

**Affiliations:** 1 Department of Genetics, University of Delhi South campus, Benito Juarez Road, New Delhi, India; 2 Department of Psychiatry, Dr. RML Hospital, New Delhi, India; 3 Department of Psychiatry, Western Psychiatric Institute and Clinic, University of Pittsburgh School of Medicine, PA 15213, USA

## 

Schizophrenia (SZ) is a debilitating neuropsychiatric disorder with ~80% heritability. Despite several genetic studies including linkage and candidate gene association and more recently GWAS, which have identified several risk variants, the total heritability of SZ remains elusive. In addition, a number of gene expression studies have reported dysregulation of candidate genes both in brain and blood of SZ cases compared to controls. Although, the role of coding, promoter, intergenic and UTR SNPs, have been demonstrated, very little is known about the role of miRNA binding site SNPs. In this study, we undertook to investigate the association, if any, of this important class of regulatory variants with SZ. Using in silico prediction tools, 27 functionally relevant SNPs from around 150 candidate genes were prioritized and genotyped in a north Indian SZ cohort (n=507 cases; n=522 controls).

Test of association of these SNPs showed only one variant rs7430 in PPP3CC to be associated (p=0.01) with SZ. Analysis of genotype data in a subset of patients (TD positive n=89; TD negative n=160) with Tardive dyskinesia (TD), an iatrogenic disorder of SZ, showed association of rs4846049 in MTHFR (p=0.04) & rs17881908 in GCLM (p= 0.05 ) with this condition. Further regression analysis of the genotype data with neurocognitive measures in a subset (cases n=152; controls n=290) of the study cohort, showed significant association of nine SNPs (p< 0.05) with different domains of cognition. Based on this moderately powered study, the contribution of miRNA binding site SNPs in candidate genes to SZ and to TD seems negligible. However, their promising contribution to cognitive parameters warrants additional investigations.

